# UP1306: A Composition Containing Standardized Extracts of *Acacia catechu* and *Morus alba* for Arthritis Management

**DOI:** 10.3390/nu11020272

**Published:** 2019-01-26

**Authors:** Mesfin Yimam, Teresa Horm, Laura Wright, Ping Jiao, Mei Hong, Lidia Brownell, Qi Jia

**Affiliations:** 1 Unigen Inc., 2121 South State Street, Suite 400, Tacoma, WA 98405, USA; thorm@unigen.net (T.H.); pjiao@unigen.net (P.J.); meih@unigen.net (M.H.); lbrownell@unigen.net (L.B.); qjia@unigen.net (Q.J.); 2 Fred Hutch Cancer Research, 1100 Fairview Ave N, Seattle, WA 98109, USA; lwright@fredhutch.org

**Keywords:** collagen induced arthritis, *Acacia catechu*, *Morus alba*, UP1306

## Abstract

Osteoarthritis (OA) is characterized by progressive articular cartilage degradation. Although there have been significant advances in OA management, to date, there are no effective treatment options to modify progression of the disease. We believe these unmet needs could be bridged by nutrients from natural products. Collagen induced arthritis in rats was developed and utilized to evaluate anti-inflammatory and cartilage protection activity of orally administered botanical composition, UP1306 (50 mg/kg) and Methotrexate (75 µg/kg) daily for three weeks. Objective arthritis severity markers, urine, synovial lavage, and serum were collected. At necropsy, the hock joint from each rat was collected for histopathology analysis. Urinary cartilage degradation marker (CTX-II), pro-inflammatory cytokines (tumor necrosis factor α (TNF-α), interleukin-1β (IL-1β), and IL-6), and proteases (Matrix Metallopeptidase 3 (MMP3) and 13) were measured. Rats treated with UP1306 showed statistically significant improvements in arthritis severity markers, including uCTX-II (91.4% vs. collagen-induced arthritis (CIA)), serum IL-1β, TNF-α, and IL-6 levels as well as synovial MMP-13. The histopathology data were also well aligned with the severity score of arthritis for both UP1306 and Methotrexate. UP1306, a botanical composition that contains a standardized blend of extracts from the heartwood of *Acacia catechu* and the root bark of *Morus alba*, could potentially be considered as a dietary supplement product for the management of arthritis.

## 1. Introduction

Osteoarthritis (OA) is characterized by progressive articular cartilage degradation that will lead to joint pain, immobility, and other functional impairments [1]. Arthritis affects an estimated 54.4 million adults in the US, with projections of 78.4 million by the year 2040 [2]. Although there have been significant advances in OA management, to date, there are no effective treatment options to modify progression of the disease. The current approach mainly targets to relieve symptoms by reduction of pain and improvement of joint function using oral or topical over-the-counter nonsteroidal anti-inflammatory drugs (NSAIDs). This strategy is known to mask the primary cause leading to irreversible damage to the articular structure. Chronic uses of NSAIDs for symptomatic relief of OA are also limited due to their gastrointestinal, renal, and cardiovascular side effects [3]. Hence, these unmet needs could be bridged with natural products.

It has been a common practice to standardize the extract of a medicinal plant to be used alone or in combination with other functional bioactives or nutrients for multiple human ailments. For instance, in traditional and contemporary medicine, *Morus alba* root-bark extract has been reported to have antibacterial [4], antioxidant, hypoglycemic [5,6], hypolipidemic, neuroprotective, antiulcer, analgesic [7,8,9], and anti-inflammatory activities [10]. Extracts and prenylated flavonoids from Morus are known to inhibit nitric oxide and interleukin-6 (IL-6) production, downregulate inducible nitric oxide synthase [11], inhibit activation of Nuclear Factor kappa light chain enhancer of activated B cells (NF-κB) [12], and inhibit a tumor necrosis factor α (TNF-α), [13] and interleukin-1β (IL-1β) production [14]. This suggests its use in inflammatory conditions. Similarly, *Acacia catechu* extract has been widely used for its anti-oxidation [15], free radical scavenging [16], DNA damage protection [17], antiproliferative, cytotoxic [18], antidiabetic [19,20], hepatoprotective [21], analgesic [22,23], chemoprotective [24], anti-microbial [25], and anti-inflammatory activities [26]. 

These properties of A. catechu and M. alba were indeed translated into beneficial applications for OA when their standardized composition, UP1306, was tested in vitro and in vivo. To mention a few tests, UP1306 was found to cause (a) suppression of inflammation and pain sensitivity in carrageenan induced rat paw edema model [27], (b) modulation of cyclooxygenase and lipoxygenase activities [27], (c) synergistic inhibition of glycosaminoglycan release ex vivo [27], and (d) increased cartilage sparing activities in monoiodoacetate-induced rat OA model [28]. In a randomized and double-blinded placebo-controlled clinical trial, UP1306 administered at 400 mg/day to arthritic subjects showed significant reduction in urinary C-telopeptides of type II collagen (CTX-II), when compared to placebo after 12 weeks of daily supplementation [29]. In each of these studies, the effect of UP1306 on pro-inflammatory cytokines and matrix degrading enzymes were implied, although it was not directly measured. Herein, we designed a study that utilizes the collagen induced arthritis model to address these gaps. 

The collagen induced arthritis model is known to cause autoimmunity to type-II collagen that could lead to autoimmune arthritis which encompass inflammation of synovial joint, cartilage destruction, and bone erosion [30]. Both cellular and humoral immunity are involved in the pathogenesis of the disease. The pro-inflammatory cytokines interleukin-1 (IL-1), IL-6, and TNF-α are heavily involved in the etiology of arthritis [31]. It has been known that TNF-α has an early and crucial role in the cascade of pro-inflammatory cytokine production and subsequent inflammatory process. Previous studies showed increase in arthritis severity when TNF-α works in synergy with IL-1β. With the concept of TNF-α as the tip of pro-inflammatory network in early Rheumatoid Arthritis (RA) pathogenesis, anti-TNF-α antibodies (e.g., infliximab, etanercept, and adalimumab) were developed as prescription drugs for the treatment of rheumatoid arthritis by neutralizing TNF-α [32]. Those biologics showed remarkable clinical benefit validating the hypothesis that TNF-α plays a major role in the pathology of RA. While patients receiving anti-TNF therapy have shown significant improvement in arthritic signs and symptoms, not all patients were equally responsive for anti-TNF therapy indicating the need for additional cytokine inhibitions, such as IL-6 and IL-1β [33]. Similar efficacies have also been achieved with IL-6 and IL-1β inhibitors (e.g., Tocilizumab and canakinumab, respectively) for RA patients [34]. These pro-inflammatory cytokines play key roles in disease initiation and progression by triggering other inflammatory cytokines and inducing cartilage degrading enzymes, such as metalloproteinases and aggrecanases [35]. Considering its application in arthritis, commonly used natural compounds, such as curcumin, Boswellia extracts, and others, have employed this model to address mechanic and functional based activities of products [36,37]. Considering the collagen induced arthritis as a typical model for rheumatoid arthritis, we used Methotrexate as a reference compound in our study. It is an anti-neoplastic immunosuppressant drug that is widely used for treating rheumatoid arthritis at lower dosages. Its low cost, extensive safety record, and weekly treatment regimen makes it an attractive option in early RA.

In the present study, we carried out collagen induced rat arthritis model to further investigate the mechanism, where UP1306 imposes its symptomatic relief and cartilage protection activities in arthritis. Key pro-inflammatory cytokines and matrix degrading enzymes were used as objective measures for data interpretations.

## 2. Materials and Methods

### 2.1. Composition

The Composition, UP1306, was prepared by mixing the standardized aqueous extract (that contains at least 65% catechins) from heartwood of A. catechu and M. alba root bark ethanol extract (that contains not less than 7% stilbenes and bioflavonoids) at a ratio of 1:2 by weight. The active contents in UP1306 are catechins (at least 15%), stilbenes, and bioflavonoids (at least 2%).

### 2.2. Model Induction and Treatment

Male Sprague Dawley rats (7–8 weeks old, *n* = 40) were purchased from Charles River Laboratories Inc. (Wilmington, MA, USA) and acclimated upon arrival for two weeks before being assigned randomly to their respective treatment groups: G1 = Normal control (−) (*n* = 10/group), G2 = collagen-induced arthritis (CIA) + Vehicle (0.5% Carboxy Methylcellulose) (*n* = 10/group), G3 = CIA + Methotrexate (+) (75 µg/kg) (*n* = 10/group), and G4 = CIA + UP1306 (+) (50 mg/kg) (*n* = 10/group). Treatment was initiated two weeks before model induction and lasted for an additional three weeks thereafter. Collagen type-II (Lot # 845) from bovine nasal septum and Incomplete Freund’s adjuvant (IFA) (Lot # SLBR0642v) were purchased from Elastin Products Company (Owensville, MI, USA) and Sigma (St. Louise, MO, USA), respectively. All materials were kept at suitable temperature as recommended by the manufacturer. At the time of preparation, 60 mg of collagen was weighed and added to a pre-chilled 15 mL 0.1 M acetic acid in a 60 mL-sized flask with a magnetic stirrer to yield 4 mg/mL concentration [38,39]. The mixture was dissolved by gently stirring overnight at 4 °C. The next morning, the dissolved collagen was emulsified with equal volume of IFA (15 mL) to achieve a final concentration of 2 mg/mL Collagen. Rats sedated with isoflurane were then primed intradermally with 400 µL of the emulsified collagen at the base of their tail at two sites using a 1 mL syringe fitted with a 26 g needle. The dissolved mixture was kept in an ice bucket and stirred between groups at the time of injection to preserve uniform consistency. On the seventh day, rats were inoculated with a booster dose of 2 mg/mL type II collagen emulsified with equal volume of incomplete adjuvant at 100 µL/rat/site. 

### 2.3. Clinical Observation

Clinical findings such as arthritis severity index, paw thickness, ankle diameter (using Digital Absolute, Model # PK-0505CPX, Mitutoyo Corporation, Kawasaki, Japan), and pain sensitivity (using Randall Selitto, IITC Life Science Inc., Woodland Hills, CA, USA) were monitored during the course of study. Urine was collected from overnight fasted rats using metabolic cages after three weeks of treatment post model induction. At Necropsy, serum from the cardiac and synovial lavage (100 μL of saline was injected into the articular cavity and aspirated back to the syringe) for biomarkers and ankle joint for histopathology were collected from each animal. 

### 2.4. Histopathology

For histopathological examination, the ankle joints were kept in 10% formalin for 72 h. The fixed specimens were then decalcified with Calci-Clear Rapid for one and a half days and embedded in paraffin. Standardized 5 μm serial sections were obtained at the medial and lateral section in the sagittal plane of the joint and were stained with hematoxylin and eosin (HE) and Safranin O-fast green to enable evaluation of proteoglycan content. A modified Mankin system [40] was used to score structural and cellular alterations of joint tissues resulting from disease progression and/or treatment efficacy. The histological analysis was conducted at Nationwide Histology and slides were examined by a certified pathologist.

### 2.5. Assays

#### 2.5.1. Urine CTX-II-

Rat urine samples were diluted 1:3 and the presence of CTX-II was measured using the Rat CTX-II ELISA kit from Mybiosource (product#: MBS2880519) as follows: diluted urine was added to a microplate coated with CTX-II antibody and allowed to bind for 2 h at 37 °C. A biotin-conjugated antibody against CTX-II was then added and allowed to bind to the CTX-II from the rat urine for 1 h at 37 °C. The microplate was washed thoroughly to remove unbound urine and antibody, before an enzyme-conjugated avidin antibody was added to bind to the biotin-conjugated antibody for specific detection. The avidin antibody was allowed to bind for 1 h at 37 °C. Washing was repeated, enzyme substrate was added, and the plate was developed for 30 min at 37 °C. After the addition of stop solution, the absorbance was read at 450 nm, multiplied by dilution factor, and the concentration of CTX-II calculated based on the absorbance readings of a CTX-II standard curve. CTX-II amount was normalized to the amount of Creatinine in the urine using the Creatinine Parameter Assay Kit from R and D Systems (product#: KGE005) as follows: urine was diluted 1:20, mixed with alkaline picrate (5 parts 0.13% picric acid: 1 part 1 N NaOH) in a microplate and incubated at room temp for 30 min. Absorbance was read at 492 nm and Creatinine amount in urine was calculated based on the absorbance readings of a Creatinine standard curve.

#### 2.5.2. Serum IL-1β, TNF-α and IL-6 ELISA

Rat serum was diluted 1:3 (IL-1β) or 1:2 (TNF-α and IL-6). Dilutions were selected based on the ELISA Kit manufactures suggestions for detection of protein level in the serum and the amount of serum available for each study subject. The presence of IL-1β, TNF-α, or IL-6 was measured using the Rat IL-1β, TNF-α, or IL-6 Quantikine ELISA kit from R and D Systems (product#: RLB00 for IL-1β, RTA00 for TNF-α, and R6000B for IL-6) as follows: diluted serum was added to a microplate coated with polyclonal IL-1β, TNF-α, or IL-6 antibody and allowed to bind for 2 h at room temperature. The microplate was washed thoroughly to remove unbound serum and then a polyclonal enzyme-conjugated IL-1β, TNF-α, or IL-6 antibody was added and allowed to bind for 2 h at room temperature. Washing was repeated, enzyme substrate was added, and the plate was developed for 30 min at room temperature. After the addition of stop solution, the absorbance was read at 450 nm, multiplied by dilution factor, and the concentration of IL-1β/TNF-α /IL-6 calculated based on the absorbance readings of an IL-1β/TNF-α /IL-6 standard curve. IL-1β/TNF-α /IL-6 amount was normalized to the amount of total protein in serum using the Pierce BCA Protein Assay kit from ThermoFisher Scientific (product#: 23225) as follows: serum was diluted 1:50, mixed with bicinchoninic acid (BCA) reagent in a microplate, and incubated at 37 °C for 30 min. Absorbance was read at 580 nm and protein concentration in serum was calculated based on the absorbance readings of a bovine serum albumin standard curve. 

#### 2.5.3. Synovial MMP-13 and Serum MMP-3 ELISA

Rat synovial fluid or serum was diluted either 1:2, 1:3, or 1:4. Dilutions were selected based on the ELISA Kit manufactures suggestions for detection of the level of protein in the serum and the amount of serum available for each study subject). The presence of MMP-13 or MMP-3 was measured using the Rat Matrix Metallo Proteinase 13 or 3 (MMP-13 or 3) ELISA kit from Mybiosource (product#: MBS702112 for MMP-13 and MBS729026 for MMP-3) as follows: diluted synovial fluid or serum was added to a microplate coated with MMP-13 or MMP-3 antibody and allowed to bind for 2 h at 37 °C. The samples were removed and then a biotin-conjugated MMP-13 or MMP-3 antibody was added and allowed to bind for 1 h at 37 °C. The microplate was thoroughly washed and an avidin-conjugated Horse Radish Peroxidase was added and allowed to bind for 1 h at 37 °C. Enzyme substrate was then added and the plate was developed for 30 min at 37 °C. After the addition of stop solution, the absorbance was read at 450 nm, multiplied by dilution factor, and the concentration of MMP-13 or MMP-3 calculated based on the absorbance readings of an MMP-13 or MMP-3 standard curve. MMP-13 or MMP-3 amount was normalized to the amount of total protein in synovial fluid using the Pierce BCA Protein Assay kit from ThermoFisher Scientific (product#: 23225) as follows: synovial fluid was diluted 1:20, mixed with bicinchoninic acid (BCA) reagent in a microplate, and incubated at 37 °C for 30 min. Absorbance was read at 580 nm and protein concentration in synovial fluid was calculated based on the absorbance readings of a bovine serum albumin standard curve. 

All animal experiments were conducted according to institutional guidelines congruent with the guide for the care and use of laboratory animals.

### 2.6. Statistical Analysis

Data were analyzed using Sigmaplot (Version 11.0, San Jose, CA, USA). The results are represented as mean ± one SD. Statistical significance between groups was calculated by means of single factor analysis of variance followed by a paired *t*-test. *p*-values less than or equal to 0.05 (*p* ≤ 0.05) were considered as statistically significant. When normality test failed, for nonparametric analysis, data were subjected to Mann-Whitney sum ranks for *t*-test and Kruskal-Wallis one-way analysis of variance on ranks for ANOVA. Linear trapezoid rule was used to calculate AUC for days 12–18. For example, AUC (d12–d13) = {(Mean value d12 + Mean value d13)/2} × (Mean value d13 − Mean value d12). Percent inhibition is calculated as % Inhibition = {(Mean value of treatment-mean value of CIA+)/(Mean value of control-mean value of CIA)} × 100. 

## 3. Results

### 3.1. Arthritis Severity Index

**Figure 1:**
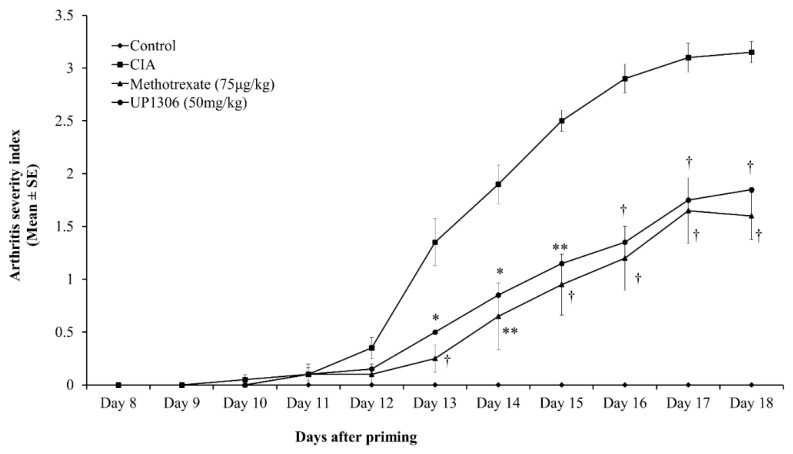
Changes of Arthritis severity index: Rats (*n* = 10/group) were orally treated with UP1306 (50 mg/kg) and Methotrexate (75 µg/kg) daily for three weeks post model induction. Score 0: No sign of grossly visible arthritis (Normal); 1: Swelling and/or redness of one or two interphalangeal joints (or mild swelling and erythema of digits or ankles); 2: Involvements of three to four interphalangeal joints (or moderate swelling and erythema of digits or ankles); 3: Swelling of entire paw (or marked swelling of paws including digits); 4: Deformity and ankylosis (or sever swelling and erythema with limited motion in many joints). Data are mean ± SE. * *p* ≤ 0.05; ** *p* ≤ 0.001; † *p* ≤ 0.0001 vs. CIA.

Rats continued to show a slow progression of disease for the duration of the study. As seen in Figure 1, rats treated with Methotrexate and UP1306 showed statistically significant suppression in arthritis severity from day 13 and continued this significance for the duration of the study. At the end of the study, average severity scores of 3.15 ± 0.32, 1.85 ± 0.74, and 1.6 ± 0.70, were observed for CIA rats treated with Vehicle, UP1306, and Methotrexate, respectively. When the area under the arthritis severity curve was calculated, percent reductions of 58.9% (*p* = 0.01) and 51.1% (*p* = 0.02) with statistical significance were observed from Methotrexate and UP1306 treatment (Table 1).

### 3.2. Ankle Diameter

**Figure 2:**
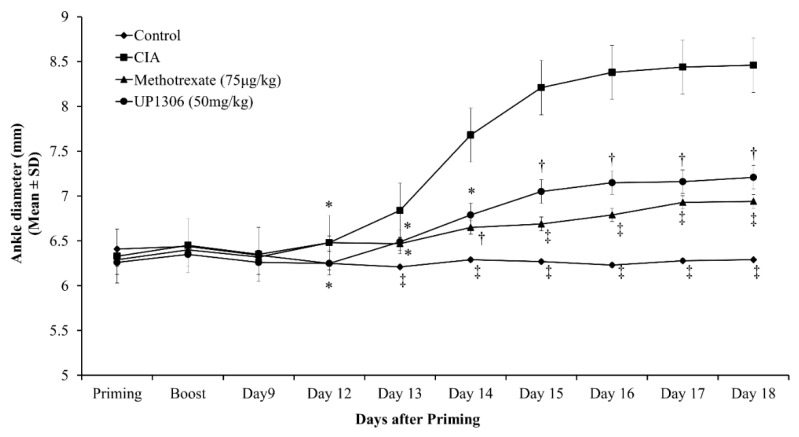
Changes of rats’ ankle width as a measure of arthritis severity in CIA model. Rats (*n* = 10/group) were orally treated with UP1306 (50 mg/kg) and Methotrexate (75 µg/kg) daily for three weeks post model induction. Data are mean ± SE. * *p* ≤ 0.05; † *p* ≤ 0.0001; ‡ *p* ≤ 0.00001 vs. CIA.

A similar pattern in the reduction of ankle diameter was observed for rats treated with Methotrexate and UP1306 for the duration of the study (Figure 2). These groups showed a statistically significant reduction in ankle width when the area under the curve was considered for days 12 to 18. Percent reductions of 71.6% and 59.7 % with statistical significance in paw edema were observed for rates treated with Methotrexate and UP1306, respectively (Table 1).

### 3.3. Paw Thickness

**Figure 3:**
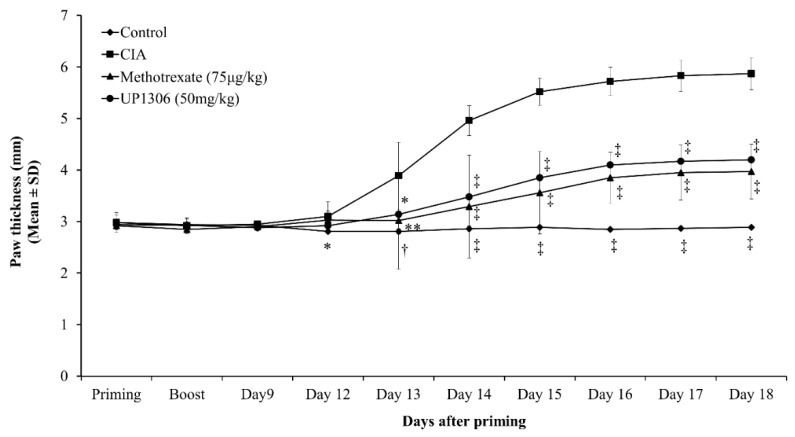
Changes in rat paw thickness in CIA model. Rats (*n* = 10/group) were orally treated with UP1306 (50 mg/kg) and Methotrexate (75 µg/kg) daily for three weeks post model induction. Data are mean ± SE. * *p* ≤ 0.05; ** *p* ≤ 0.001; † *p* ≤ 0.0001; ‡ *p* ≤ 0.00001 vs. CIA.

In agreement with the severity score and ankle diameter, rats treated with Methotrexate and UP1306 showed a statistically significant reduction in paw swelling starting from day 13; this significance was maintained for the duration of the study (Figure 3). When the total area under the swelling curve (day 12–day 18) was considered, Methotrexate and UP1306 groups showed statistically significant reductions (69.6% and 61.1%) in paw edema compared to the vehicle treated CIA group, respectively (Table 1). 

### 3.4. Pain Sensitivity

**Figure 4:**
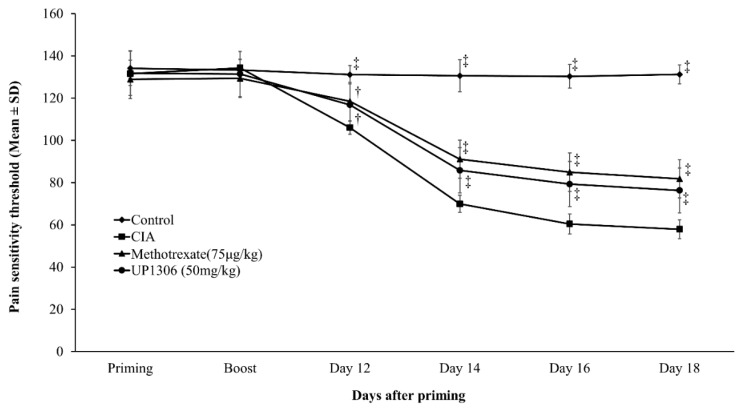
Pain tolerance of rats compared to vehicle treated CIA rats. Rats (*n* = 10/group) were orally treated with UP1306 (50 mg/kg) and Methotrexate (75 µg/kg) daily for three weeks post model induction. Data are mean ± SD. † *p* ≤ 0.0001 vs. vehicle treated CIA; ‡ *p* ≤ 0.00001 vs. vehicle treated CIA.

Response to pressure as a measure of pain sensitivity was measured using the Randall–Selitto probe attached to an electronic monitor on priming day, boost day, and days 12, 14, 16, and 18. Both the left and right hind legs were monitored on those days and their average was used for data analysis. Changes from the vehicle-treated CIA rats have been reported as pain tolerance on those days. The highest pain tolerance was observed for rats in the Methotrexate group followed by the UP1306 group (Figure 4). These reductions—such as 11.8%, 30.3%, 40.6%,and 41.6% for Methotrexate and 10.2%, 22.7%, 31.3%, and 31.3% for UP1306 on days 12, 14, 16, and 18, respectively—were statistically significant as of day 12 and remained significant for the rest of the duration of the study.

### 3.5. Serum Pro-Inflammatory Cytokines

**Figure 5:**
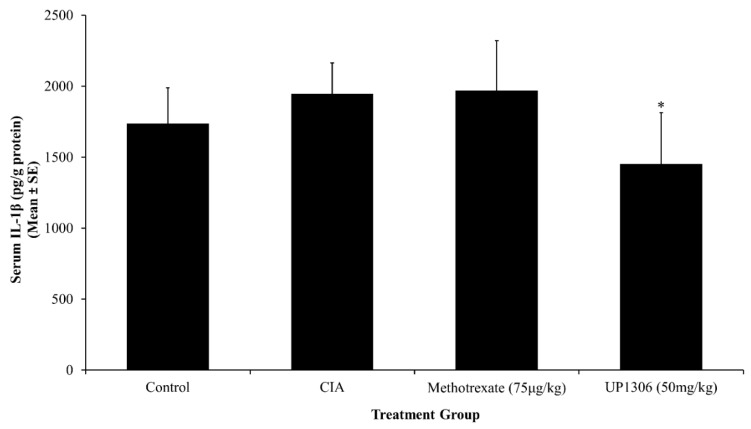
Effect of UP1306 on rat serum interleukin-1β (IL-1β) level normalized with protein in CIA model treated for three weeks post immunization. Data are Mean ± SE. * *p* ≤ 0.05 vs. vehicle treated CIA.

**Figure 6:**
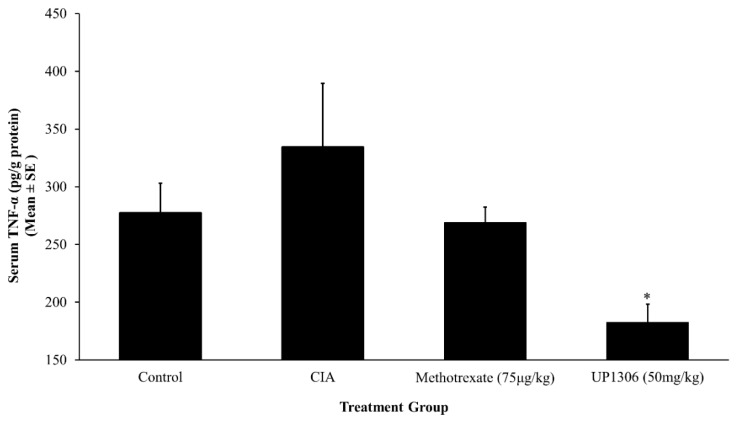
Effect of UP1306 on rat serum tumor necrosis factor α (TNF-α) level normalized with protein in CIA model treated for three weeks post immunization. Data are Mean ± SE. * *p* ≤ 0.05 vs. vehicle treated CIA.

**Figure 7:**
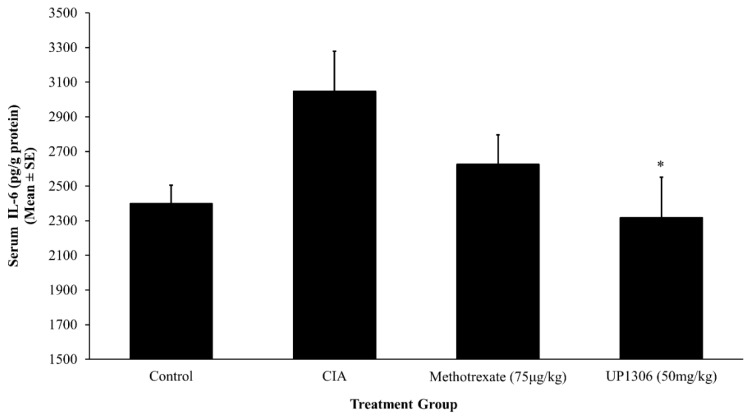
Effect of UP1306 on rat serum IL-6 level normalized with protein in CIA model treated for three weeks post immunization. Data are Mean ± SE. * *p* ≤ 0.05 vs. vehicle treated CIA.

An increased production of cytokines is the integral part of collagen induced arthritis pathology. Rats treated with UP1306 showed statistically significant reduction in serum IL-1β level when compared to the vehicle treated CIA group (Figure 5). There was a slight increase in serum IL-1β for the rats treated with Methotrexate. Similarly, marked changes in serum TNF-α and IL-6 levels were observed. As depicted in Figure 6 and Figure 7, significant increases in serum TNF- α and IL-6 level were observed for the vehicle-treated CIA group compared to the normal control. UP1306-treated rats showed statistically a significant reduction in serum IL-6 (112.4% inhibitions, compared to diseased control) and TNF-α (266.6% inhibitions, compared to diseased control) levels when compared to vehicle-treated diseased rats (Figure 6 and Figure 7). Methotrexate-treated rats showed modest but not statistically significant reductions in serum IL-6 (64.9% inhibitions, compared to a diseased control) and TNF-α (115.3% inhibitions, compared to diseased control) (Figure 6 and Figure 7).

### 3.6. Synovial MMP-13

**Figure 8:**
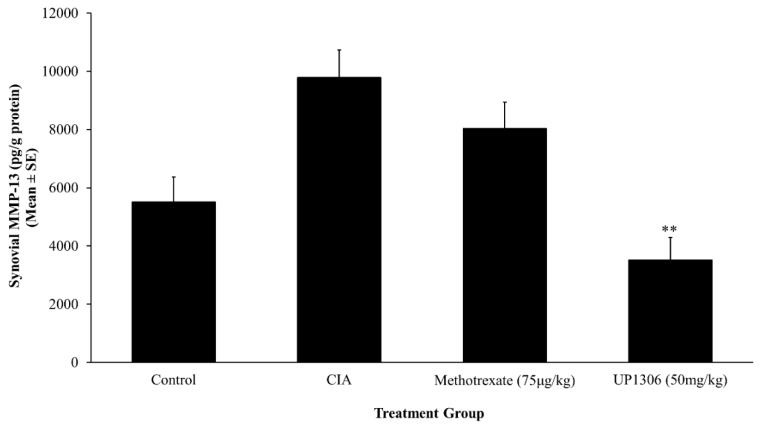
Synovial MMP-13 level from CIA rats. Rats (*n* = 10/group) were orally treated with UP1306 (50 mg/kg) and Methotrexate (75 µg/kg) daily for three weeks post model induction. Data are mean ± SE. ** *p* ≤ 0.001vs vehicle treated CIA.

A marked increase in the level of synovial MMP-13 level was observed for the vehicle-treated diseased rats, when compared to the normal control rats. As seen in Figure 8, rats treated with UP1306 showed a statistically significant reduction in MMP-13 level compared to vehicle-treated CIA rats. This inhibition for UP1306-treated rats was calculated as 146.7% vs. CIA treated rats. A moderate and non-significant reduction in synovial MMP-13 level was observed for the Methotrexate group. 

### 3.7. Serum MMP-3

**Figure 9:**
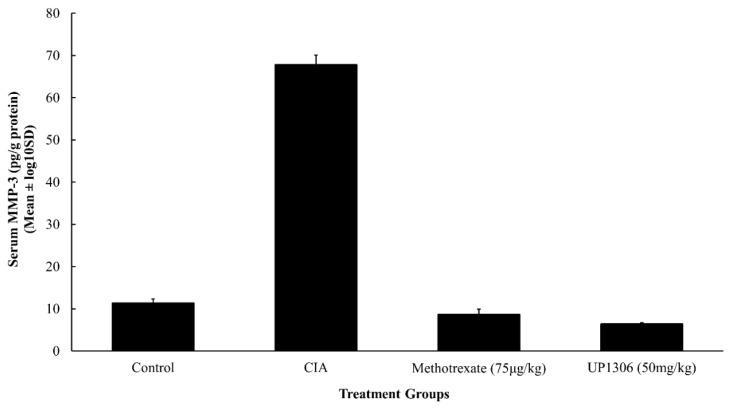
Synovial MMP-3 level from CIA rats. Rats (*n* = 10/group) were orally treated with UP1306 (50 mg/kg) and Methotrexate (75 µg/kg) daily for three weeks post model induction. Data are Mean ± log10SD.

As seen in Figure 9, an increased level of MMP-3 was observed for vehicle-treated CIA rats, when compared to the normal control. On the other hand, percent inhibitions of 104.7% and 108.8% were observed for rats treated with Methotrexate and UP1306, respectively. Neither the increase in the CIA (compared to the normal control) rats nor the decreases observed in the treated rats (compared to the CIA rats) were statistically significant. There were bigger variations within the individual rats in the vehicle-treated CIA rats which could have an impact on the absence of statistical significance.

### 3.8. Urinary CTX-II

**Figure 10:**
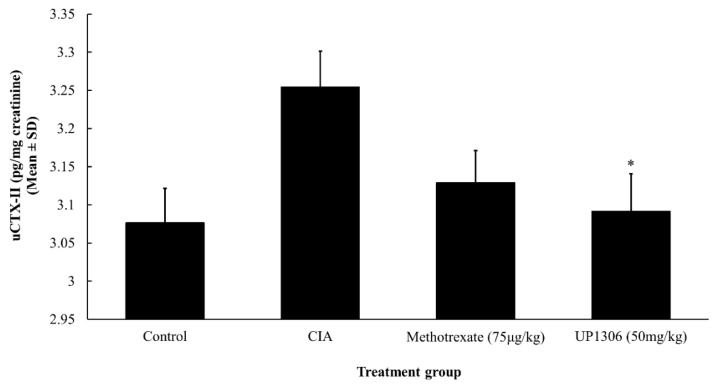
Effect of UP1306 on rat urinary CTX-II level normalized with creatinine in CIA model. Rats (*n* = 10/group) were orally treated with UP1306 (50 mg/kg) and Methotrexate (75 µg/kg) daily for three weeks post model induction. Data are mean ± SD. * *p* ≤ 0.05 vs. vehicle treated CIA.

Significant urine CTX-II level changes both in disease inductions and treatment effects were observed in this study. As illustrated in Figure 10, a statistically significant increase in urine CTX-II level was observed for the vehicle-treated CIA group compared to the normal control confirming severity of disease. Treatment with UP1306 spared significant degradation of cartilage (up to 91.4%) compared to vehicle-treated diseased CIA rats. The positive control Methotrexate showed 70.4% inhibition in cartilage degradation compared to CIA with *p* = 0.06. 

### 3.9. Histopathology Findings

**Figure 11:**
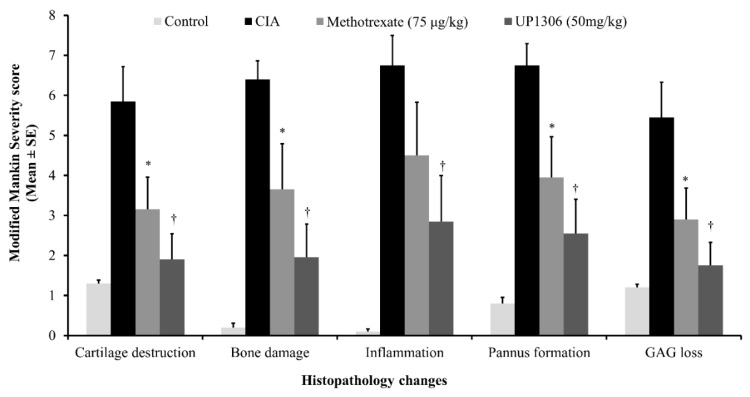
Histopathology findings of rats’ ankle joint treated with UP1306 in CIA model. Cartilage destruction (0–6): Cartilage thickness/thinning, irregular surface frayed/fissure loss, degeneration, ulcerative necrosis/fragmentation, severe disorganization/chaotic; Bone damage (0–6): Subchondral bone thickness/volume and density, osteoclastic activity, subchondral bone damage; Inflammation/Cellular infiltration (0–6): Cellular Infiltration/Inflammation and Proliferation, hypercellular, cluster/hypocellular; Pannus formation (0–6): Fibrovascular Proliferation replacing periarticular/capsule/bone (Pannus), condyle and/or tibial plateau, menicus reduction, fusion, adhesion; Matrix GAGs loss (0–6): Matrix GAG reduction: radial, interterritorial to pericellular loss of staining, femoral condyle/tibial plateau integrity, and thickness of articular Cartilage. Data expressed as Mean ± SE. * *p* ≤ 0.05; † *p* ≤ 0.001.

**Figure 12:**
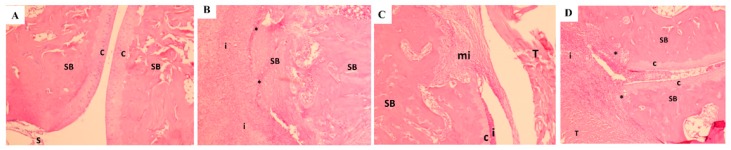
Microscopic changes in HE staining: (**A**). Normal Control + Vehicle: HE 50×–Histology of the hock with minimal pathology. SB–subchondral bone (pink), C–cartilage (purple), S–synovium; (**B**). CIA + Vehicle: 50X HE: Inflammation (i) and diffuse necrosis of the subchondral bone (SB) with effacement of the articular surfaces (*). There is complete loss of the bone architecture; (**C**). CIA + Methotrexate: 50×-HE–These images demonstrate an area of fibroplasia with monocytic inflammation (mi). SB–subchondral bone, C–cartilage with adhered inflammation (i), T–Tendon; (**D**). CIA + UP1306: 50× HE–Subchondral bone (SB) with the overlying cartilage (c) and adjacent bone being effaced (*) by inflammation; (i) T–tendon expanded by edema and infiltrated by inflammation.

**Figure 13:**
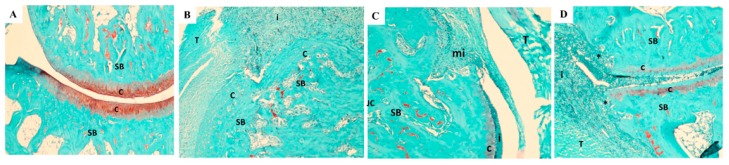
Microscopic changes in safranin staining. (**A**). Normal Control + Vehicle: Safranin 50×– Histology of the hock with minimal pathology. SB–subchondral bone (green), c–cartilage (red orange); (**B**). CIA + Vehicle: 50× Safranin: Diffuse necrosis of cartilage (c) and subchondral bone (SB). i–inflammation, T–tendon. The lack of red-orange staining on articular surfaces highlights the cartilage loss. Expanded by edema and infiltrated by inflammation; (**C**). CIA + Methotrexate: × Safrarin; These images demonstrate an area of fibroplasia with monocytic inflammation (mi). SB–subchondral bone, C–cartilage with adhered inflammation (i), T–Tendon; JC: joint capsule lined by synovium. (**D**). CIA + UP1306: 50× Safranin–Subchondral bone (SB) with the overlying cartilage (c) and adjacent bone being effaced (*) by inflammation; (i) T–tendon expanded by edema and infiltrated by inflammation.

The histopathology data was in alignment with the severity score of arthritis. When compared to the normal control rats, vehicle-treated rats showed severe synovitis, marked cartilage degradation, synovial hyperplasia, pannus formation, and bone erosion (Figure 11, Figure 12 and Figure 13). In contrast, rats treated with UP1306 had nearly normal morphology, with minimal alteration in matrix integrity, a smoother articulation cartilage surface, low levels of mononuclear cell infiltration, and synovial hyperplasia, as well as reduced articular bone damage. Except for inflammation, Methotrexate-treated animals also showed similar efficacy to that of the UP1306 group. 

## 4. Discussion

The CIA model in rats is the most commonly studied autoimmune model of RA with several pathological features resembling the immune-mediated polyarthritis in dogs and humans [41]. Its short duration between immunization and disease manifestations makes the model feasible for therapeutic efficacy evaluations. Following inoculation of heterogenic type II collagen (CII), rats mount both humoral and cellular responses to the antigen [38]. This sensitization subsequently leads to the host animal attacking its own type II collagen, which is predominantly present in the joint cartilage and hence results in erosive or non-erosive joint destruction. The pathophysiology of the disease is highly orchestrated and complex. Upon induction, rats will experience inflammatory pain and swelling, cartilage degradation, synovial hyperplasia, pannus formation, mononuclear cell infiltration, deformity, and immobility.

In the current study, rats started to show the pathognomonic signs of arthritis on day 12 post priming followed by a progressive increase in severity that approached near plateau on days 17 to 18. These symptoms were mitigated by oral treatment of an immune suppressant–Methotrexate and also a natural polyphenol composition-UP1306. Both treatment groups (Methotrexate and UP1306) showed measurable relief in arthritis severity, swelling, ankle width, and pain sensitivity when compared to the vehicle-treated diseased rats. When data for arthritis severity, paw thickness, and ankle diameter were pooled together for the duration of the study period from day 12–18 (where visible signs of arthritis were observed), CIA rats treated with Methotrexate and UP1306 showed statistically significant reduction in all of the cardinal signs of arthritis suggesting their application for symptomatic relief of arthritis. 

TNF-α and IL-1β are the two primary cytokines involved in the initiation and progression of arthritis [42], mainly through (a) downregulation of the synthesis of major extracellular matrix components by inhibiting anabolic activities of chondrocytes [43,44]; (b) induction of additional cytokines (such as IL-6), chemokines, and extra cellular matrix degrading enzymes (MMPs and aggrecanases) [45,46]; (c) inhibition of anti-oxidant activity of the host [47]; and (d) induction of reactive oxygen species [48]. These processes facilitate maintenance of chronic inflammation and perpetual joint destruction in arthritic patients. For example, while injection of IL-lβ into the knee joints of rats caused joint inflammation and marked proteoglycan depletion [49,50], its blockade reversed the process [51,52,53]. Besides direct involvement in the inflammation process and cartilage degradation, dysregulation of IL-6 levels is also linked to the common clinical manifestations associated with rheumatoid arthritis pathology such as fever, fatigue, and weight loss [54]. Hence, modulating these pro-inflammatory cytokines at various stages of disease progression could alleviate the symptoms associated with arthritis and/or help to modify the disease. The anti-inflammation and cartilage protection activities of UP1306 observed in this study could be in part due to inhibition of these key pro-inflammatory cytokines. 

Supplementation with UP1306 for three weeks resulted in significant reductions in the level of fundamental matrix proteolytic enzymes, such as MMP-13 and MMP-3. Along with aggrecan breakdown, degradation of collagen is a central feature of arthritis [55]. Pro-inflammatory cytokines, such as TNF-α, IL-1β, and IL-6 are known to play important roles in cartilage matrix degradation in articular cartilage through a cascade of events that lead to stimulation of aggrecanase and matrix metalloproteinase production [42]. When MMP enzymes are activated, MMP-3 is the first to be activated. It then triggers other MMPs, such as MMP1, MMP9, and MMP13, which leads to further degradation and amplification of extracellular matrix components. This leads to a simultaneously increased release and accumulation of degradants in the joints and causes joint inflammation. During the course of disease pathology, the major histocompatibility complex presents these fragments to T cells and promotes the activation and release of large amounts of inflammatory cytokines, such as IL-1β and IL-6, which in turn increases other MMP expression levels in the chondrocytes and synovial fibroblasts. Consequentially, all these processes result in augmented collagenase activity and worsening of joint inflammation. MMP-3 and MMP-13 have been found in increased levels at the sites of cartilage erosion in cases of rheumatoid arthritis and osteoarthritis [56]. Previous studies have shown that these MMP levels in OA patient’s blood and synovial fluid were higher than in healthy people and the level was consistent with the extent of cartilage damage [57,58]. In fact, MMPs secreted into the synovial fluid can directly degrade the cartilage and bone composition leading to enhanced damage of surrounding articular structures [59]. In the current study, there was significant suppression of serum MMP-3 and MMP-13 levels by UP1306, which could provide a wholesome protection of cartilage degradation and improved pain relief. The reduction in MMPs observed in this study could partially be explained by (a) the effect of treatment materials in reducing the precipitating pro-inflammatory cytokines and/or (b) the activity of treatment materials by directly suppressing expression of these matrix degrading enzymes.

Urine C-terminal telopeptide of type II collagen (uCTX-II) has been by far the most studied and frequently referred biomarker of cartilage degradation that could be used for the purpose of diagnosis, determining the severity of disease or extent of disease progression, prognosis, and monitoring efficacy of treatment [60]. In clinical studies, high levels of CTX-II are a good predictor of increased risk of joint destruction [61]. Degradation and loss of articular cartilage are fundamental features of collagen-induced arthritis whereby increased CTX-II level directly correlated with the time course of paw swelling and arthritis severity. Our results were in accord with previous reports [35,62]. In the current study, substantiating the effects on paw swelling, paw thickness, arthritis severity, pro-inflammatory cytokines, and matrix degrading enzymes, rats treated with UP1306 showed significantly reduced levels of uCTX-II. These findings indicate that cartilage protection activity is one of the primary functions of UP1306 suggesting its usage in management of arthritic conditions.

Together with symptoms and biomarkers, histopathological analyses of articular cartilage, synovial membrane, and subchondral bone have been used to evaluate arthritis disease progression or to measure outcome of therapeutic interventions [63]. In the current study, significant improvements in maintenance of the articular structural integrity of rats treated with UP1306 and Methotrexate were observed. These effects were demonstrated in the histopathology data as exhibited by limited loss, degeneration, or necrosis of chondrocytes, smoother articular cartilage surface, deeper and uniform stain of intracellular matrix, and close to normal contour of the subchondral bone. The changes in magnitude of histopathological severity scores for cartilage degradation, bone damage, inflammation, reactivity, and matrix integrity were computed and were found that UP1306 treatment resulted in 86.8%, 71.8%, 58.6%, 70.6%, and 87.1% inhibitions, respectively, when compared to vehicle-treated CIA rats.

Previously, active constituents of UP1306 (i.e., catechins, prenylated flavonoids, and stilbenes) have showed to possess activities suggestive of their benefits in OA management. These include (i) the inhibition of the activities of cyclooxygenase-2 (COX-2), lipoxygenase (5-LOX), platelet phospholipase A2, and pro-inflammatory cytokines, such as TNF-α, ILs 1, 2, 6, 8, and 12 [64,65] as a result of catechin; (ii) inhibition of inflammation activities [66]; (iii) suppression effect of T-cell migration and inflammation induction [67]; (iv) inhibition of nitric oxide (NO), inducible NO synthase expression, prostaglandin E2 production, and activation of NF-κB [68]; (v) inhibition of pro-inflammatory mediators, such as IL-1β, IL-6, and COX-2 [69]; and (vi) activation of total antioxidant ability [69,70] as a result of prenylated flavonoids and stilbenes from M. alba root bark extract. 

## 5. Conclusions

Collectively, in the current study, UP1306 orally supplemented rats experienced (a) reduced inflammation as reflected by reduced arthritis index, paw thickness, paw edema, and reduced inflammatory cytokines (IL-1β, IL-6, and TNF-α), (b) decreased pain sensitivity, and (c) increased cartilage sparing activity and maintenance of articular structure as indicated by lower uCTX-II and cartilage degrading enzymes (MMP-13 and MMP-3). The aggregated effects that inhibit pro-inflammatory cytokines and cartilage matrix degrading enzymes indicate that the protective effect of UP1306 on arthritis could be partially mediated through the modulation of the immune system. These properties of UP1306 suggest its potential use as an alternative natural therapy for arthritis management.

## Figures and Tables

**Table 1:** Area under treatment curve for collagen-induced arthritis (CIA) rats treated with UP1306 and Methotrexate.

Parameters	AUC (d12–d18)			
	Control	CIA	Methotrexate (75 μg/kg)	UP1306 (50 mg/kg)
Arthritis Index	0	13.50 ± 0.80	5.55 ± 0.51 *	6.60 ± 0.50 *
Ankle diameter	35.55 ± 0.02 †	47.02 ± 0.66	40.24 ± 0.16 **	41.37 ± 0.30 *
Paw thickness	17.13 ± 0.02 †	30.41 ± 0.85	21.17 ± 0.36 **	22.30 ± 0.43 *

Linear trapezoid rule was used to calculate AUC for days 12–18. For example AUC (d12–d13) = {(Mean value d12 + Mean value d13)/2} × Mean value d13 − Mean value d12). * *p* ≤ 0.05; ** *p* ≤ 0.001; † *p* ≤ 0.0001.
